# Alum Baking Powders

**Published:** 1914-08

**Authors:** 


**Alum Baking Powders.** The referee board of the U. S. Dept. of Agriculture, consisting of Ira Remsen, Russell H. Chittenden, John H. Long, Alonzo E. Taylor and Theobald Smith, state that the amount ordinarily consumed in a day when alum baking powders are used, is 40 to 75 m.g., that in camp, subsisting mainly on baking powder biscuits, it may reach 200 m.g. In such quantities, it has no appreciable effect. Aluminum compounds added in amounts up to 1 gram are not poisonous though they cause catharsis and occasionally colic. No known baking powder is free from cathartic action if used in sufficient quantity. Hence, as was long ago taught by Witthaus, the prejudice against alum baking powders is entirely unfounded though it is wise not to use such substances immoderately.

